# Endophilin2 Interacts with GluA1 to Mediate AMPA Receptor Endocytosis Induced by Oligomeric Amyloid-*β*

**DOI:** 10.1155/2017/8197085

**Published:** 2017-07-05

**Authors:** Jifeng Zhang, Yichen Yin, Zhisheng Ji, Zhenbin Cai, Bo Zhao, Jiong Li, Minghui Tan, Guoqing Guo

**Affiliations:** ^1^Department of Anatomy, Medical College of Jinan University, Guangzhou 510630, China; ^2^Department of Neurology, Guangzhou Red Cross Hospital, Medical College, Jinan University, Guangzhou 510220, China; ^3^Department of Orthopedics, The First Affiliated Hospital of Jinan University, Guangzhou 510632, China

## Abstract

Amyloid-*β* (A*β*) plays an important role in Alzheimer's disease (AD), as oligomeric A*β* induces loss of postsynaptic AMPA receptors (AMPARs) leading to cognitive deficits. The loss of postsynaptic AMPARs is mediated through the clathrin-dependent endocytosis pathway, in which endophilin2 is one of the important regulatory proteins. Endophilin2, which is enriched in both the pre- and postsynaptic membrane, has previously been reported to be important for recycling of synaptic vesicles at the presynaptic membrane. However, the role of endophilin2 in oligomeric A*β*-induced postsynaptic AMPAR endocytosis is not well understood. In this study, we show that endophilin2 does not affect constitutive AMPAR endocytosis. Endophilin2 knockdown, but not overexpression, resisted oligomeric A*β*-induced AMPAR dysfunction. Moreover, endophilin2 colocalized and interacted with GluA1, a subunit of AMPAR, to regulate oligomeric A*β*-induced AMPAR endocytosis. Thus, we have determined a role of endophilin2 in oligomeric A*β*-induced postsynaptic AMPAR dysfunction, indicating possible directions for preventing the loss of AMPARs in cognitive impairment and providing evidence for the clinical treatment of AD.

## 1. Introduction

Alzheimer's disease (AD) is one of the main causes of cognitive disorder in the elderly [1]. Amyloid-*β* (A*β*) deposition-induced senile plaques (SP), abnormal accumulation of tau protein-induced neurofibrillary tangles, mitochondrial dysfunction, and synaptic loss are suggested to be the main mechanisms of AD pathogenesis [2, 3]. However, AD patients may demonstrate cognitive dysfunction years before the pathological changes of plaques. The soluble A*β* oligomer-induced loss of postsynaptic AMPA-type glutamate receptors (AMPA receptors (AMPARs)) is one of the important factors leading to early cognitive dysfunction in AD [4–6].

AMPARs are critical ionotropic receptors not only in excitatory postsynaptic membranes [7], participating in rapid synaptic transmission, but also in changes to synaptic strength (synaptic plasticity) [8]. Synaptic plasticity includes long-term potentiation (LTP) and long-term depression (LTD), which interact to regulate learning and memory [9]. In the postsynaptic membrane, dynamic insertion of AMPARs leads to LTP, which promotes learning and memory, whereas excessive endocytosis of AMPARs results in LTD [10, 11]. In the hippocampus and inner cortex of AD patients, the expression levels of AMPAR subunits, including GluA1, GluA2, and GluA2/3, are substantially decreased [12–14]. This phenomenon also exists in AD transgenic mice; Almeida and colleagues revealed that in neuronal cultures from AD transgenic mice, the total expression level of GluA1 showed no change, whereas the membrane surface GluA1 significantly decreased [15]. Chang and colleagues determined that not only did the surface GluA1 decrease but also that the electrical current induced by AMPARs also reduced, leading to the impairment of LTP [6].

A*β* is the enzymatic hydrolysis product of amyloid precursor protein (APP) and includes two main forms in vivo: A*β*1-42 and A*β*1-40 [16]. A*β*1-42 constructs the core of SP, while A*β*1-40 extends the plaques. Experiments in vivo and in hippocampal slices after soluble A*β*1-42 perfusion indicate that A*β*1-42 can suppress the formation of LTP in the hippocampus, potentially leading to early decreases in cognitive function in AD [17–19]. Moreover, soluble A*β*1-42 oligomers show greater neurotoxicity than A*β*1-40 [20–22]. In hippocampal neurons of APP overexpressing transgenic mice, AMPAR-mediated excitatory postsynaptic currents are also significantly decreased [23]. The expression levels of APP and GluA2/3 have been shown to be significantly decreased in the hippocampi and olfactory cortices from AD patients, with a colocalized relationship being demonstrated [24]. Furthermore, soluble A*β* oligomers often interact with AMPARs on the surface of dendritic spines [25], inducing the loss of postsynaptic AMPARs and LTD [26–28].

A number of studies have shown that the loss of postsynaptic AMPARs is mediated through the clathrin-dependent endocytosis pathway [29]. Clathrin-dependent endocytosis is one of the important mechanisms for the internalization of nutrition, antigen, growth factors, receptors, and vesicles [30]. Vesicles and receptors, together with the plasma membrane, form the coated grid bubble. Then, the bubble is cleaved and the vesicle is endocytosed into the plasma [31, 32]. Endophilin2, which is enriched in both the pre- and postsynaptic membrane, is one of the important regulatory proteins involved in clathrin-dependent endocytosis [33]. Endophilin2 mainly distributes in the synapses in neurons, both presynaptic and postsynaptic parts [34, 35]. Until now, researches of endophilin2 focus largely on the regulation of synaptic vesicle endocytosis. And our previous findings show that knockdown of endophilin2 suppresses the endocytosis process [36]. Moreover, endophilin2 is a calcium-binding protein, involved in calcium-dependent vesicle endocytosis [37]. Endophilin2 is involved in early onset gene Arc/Arg3.1-mediated AMPAR endocytosis [35]. In the current study, we found that endophilin2 interacted with AMPAR subunit, GluA1, which may be involved in the regulation of oligomeric A*β*-induced postsynaptic AMPAR endocytosis. Thus, the role of endophilin2 in oligomeric A*β*-induced postsynaptic AMPAR removal is not well understood and we therefore have tried to provide evidence of endophilin2-mediated AMPAR endocytosis induced by A*β* oligomer administration.

## 2. Materials and Methods

### 2.1. Animals

All the experiments were conducted with 1-day-old and 1-month-old Sprague Dawley (SD) rats. All animal procedures were performed in strict accordance with the recommendations in the Guide for the Care and Use of Laboratory Animals produced by the National Institutes of Health. The protocol was approved by the Institutional Animal Care and Use Committee at Jinan University, China. All efforts were made to minimize the suffering and number of animals used.

### 2.2. Plasmids and RNA Interference

Full-length endophilin2 cDNA fragments were subcloned into pEGFP-C1 and PGEX-5X-3 plasmids (Clontech, Mountain View, CA). The cDNA of the GluA1 C-terminal was inserted into the pCMV-Tag 2A vector. All constructs were verified by sequencing. A detailed description of the methods used for constructing cDNA plasmids is available in previous reports [37, 38]. Validated endophilin2 siRNA (Endo2 siRNA) fragments (5′-GCTTCGTCATCATTTAGAT-3′) and a negative control (NC; a scrambled sequence) were synthesized by Shanghai GenePharma Co. Ltd. (Shanghai, China) and were previously approved [36].

### 2.3. Preparation of A*β*1-42 Oligomers

Amyloid *β*-peptide (1-42) (human) was purchased from TOCRIS (Tocris-Bioscience, Ellisville, MO, USA). The lyophilized powder was solubilized in 50 mM Tris Buffer to 200 *μ*M and stored at −20°C according to the manufacturer's instructions. Before use, these stock solutions were thawed and incubated at 37°C for 24 h to induce peptide aggregation and then diluted to the final concentration in culture medium as previously described [39].

### 2.4. Hippocampal Neuronal Culture and Transfection

Rat hippocampal neurons were cultured as described previously [40]. Neurons were cultured in vitro in 24-well culture plates for 8–10 days before being used to perform the transfection. Calcium phosphate was used to transfect the endophilin2-pEGFPC1 (Endo2-GFP) construct and its control, or endophilin2 siRNA (Endo2 siRNA) and its control, into the neurons. A GFP expression plasmid was cotransfected with the siRNA to mark the transfected cells. 24 to 48 hours after transfection, 1 *μ*M A*β*1-42 oligomers was added to the culture medium.

### 2.5. Recombinant Protein Expression and GST Pulldown Assay

GST-fusion protein expression and pulldown assays were performed as previously described [40]. Briefly, endophilin2-GST was transformed into Top10F′ *E. coli* cells (Invitrogen), and the expression of proteins was induced by 0.1 mM isopropyl-1-thio-*β*-D-galactopyranoside (IPTG, Roche Applied Science, Indianapolis, IN, USA). Cells were pelleted and lysed, and GST-fusion protein was isolated and purified from the supernatant using glutathione agarose beads (Pierce Biotechnology, Rockford, IL, USA). The assay buffer contained 100 mM NaCl, 20 mM Tris HCl, and 5% glycerol (pH 7.0), along with 1% Triton X-100 and a cocktail of protease inhibitors consisting of 1 *μ*M phenylmethylsulfonyl fluoride, 1 *μ*g/ml pepstatin, 1 *μ*g/ml leupeptin, 1 *μ*g/ml aprotinin, and 0.1 mg/ml benzamidine (Merck, USA). Approximately 5 *μ*g GST-fused protein was incubated with 300 *μ*g 1 month SD rat brain protein, incubated for 8 h. All pulldown assays were performed at 4°C, and the results were analyzed by Western blotting.

### 2.6. Western Blotting

Western blot analysis was performed as previously described [41, 42]. Briefly, lysates were separated using SDS-PAGE and were electrophoretically transferred to a polyvinylidenedifluoride (PVDF) membrane. Membranes were blocked in Tris-buffered saline with 5% milk and 0.05% Tween and then probed with primary antibodies at 4°C overnight. Anti-GluA1 and anti-GluA2 antibodies were purchased from Millipore (MA, USA), and goat anti-endophilin2 antibody was purchased from Santa Cruz Biotechnology (Santa Cruz, CA, USA). After washing, the membranes were incubated with HRP-conjugated secondary antibodies (Jackson ImmunoResearch, PA, USA) and visualized using enhanced chemiluminescence (ECL) reagents (Pierce Biotechnology).

### 2.7. Fluorescence Immunostaining

24 hours after the A*β* oligomer treatment, the hippocampal neurons were fixed with 4% paraformaldehyde (Sigma, USA). Immunostaining was then performed using a previously described standard protocol [43]. The primary antibodies anti-GluA1-NT (Abcam, Cambridge, MA, USA) and anti-PSD95 (Abcam) were used at a dilution of 1 : 200, and Alexa Fluor 555 donkey anti-goat IgG (H + L) and Alexa Fluor 647 donkey anti-rabbit IgG (H + L; Life Technologies, Gaithersburg, MD, USA) were used at a dilution of 1 : 800. After staining, the cells were mounted on glass slides using Fluoro-Gel II with DAPI (Electron Microscopy Sciences, Hatfield, PA, USA) and were imaged with a Carl Zeiss LSM 700 confocal microscope (Zeiss, Germany). Images were acquired with the same optical slice thickness for every channel using a 63x oil objective and a resolution of 1024 × 1024 pixels.

### 2.8. Electrophysiology

Whole-cell patch-clamp recordings of miniature excitatory postsynaptic currents (mEPSC) were obtained from transfected cultured hippocampal neurons treated with A*β* oligomers on DIV 12-13 [41, 43]. During the recordings, cells were bathed in an external solution with a pH of 7.3, containing (in mM): 128 NaCl, 5 KCl, 2 CaCl_2_, 1 MgCl_2_, 15 glucose, 20 HEPES, 1 tetrodotoxin, and 100 *μ*M picrotoxin. Recording pipettes were filled with the intracellular solution containing (in mM): 147 KCl, 5 Na_2_-phosphocreatine, 2 EGTA, 10 HEPES, 2 MgATP, and 0.3 Na2GTP. Recordings were performed at room temperature in voltage clamp mode, at a holding potential of −70 mV, using a Multiclamp 700 B amplifier (Molecular Devices, Sunnyvale, CA, USA) and Clampex 10.5 software (Axon Instruments, Union City, CA, USA). The series resistance was below 30 MΩ, and data were acquired at 10 kHz and filtered at 1 kHz.

### 2.9. Statistical Analysis

Data are presented as the mean ± SEM. Statistical significance of the differences between two groups was analyzed using Student's 𝑡-test, and comparisons between more than two groups were performed using one-way ANOVA with Newman-Keuls post hoc tests. A value of *P* < 0.05 was considered to be statistically significant.

## 3. Results

### 3.1. Endophilin2 Does Not Affect Constitutive AMPAR Endocytosis

**Figure 1:**
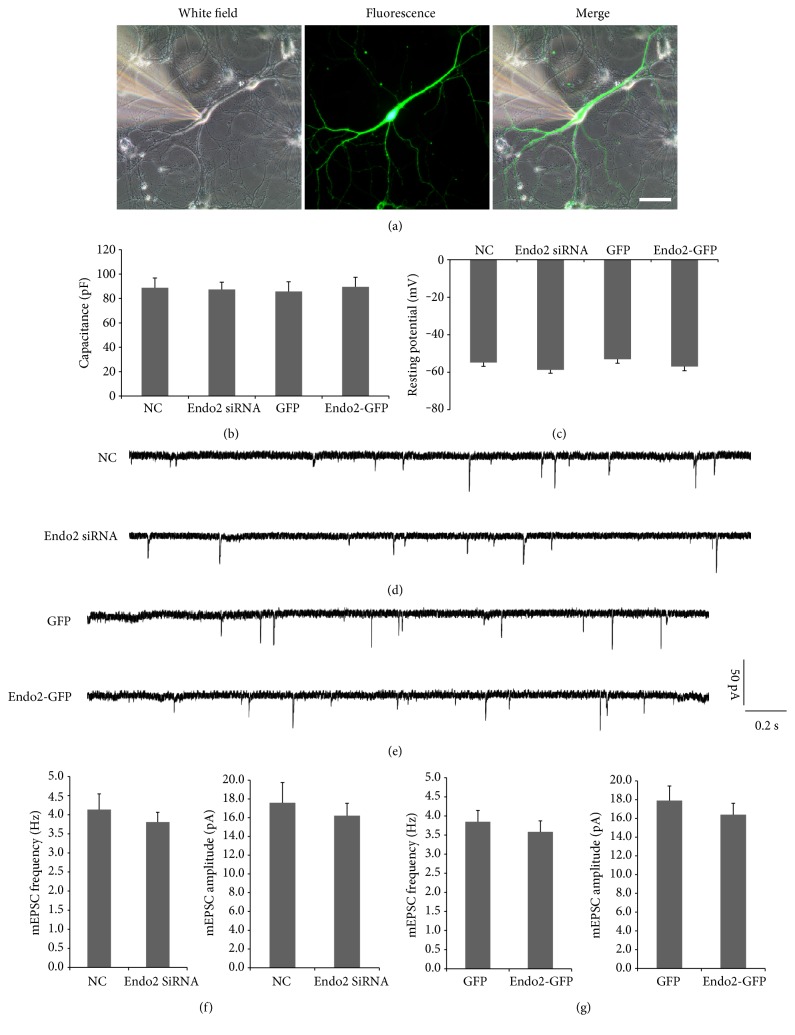
The electrophysiology of neurons with endophilin2 knockdown or overexpression. (a) Image of a neuron obtained from patch recording. Scale bar, 50 *μ*m. (b-c) Bar plots of the mean values of resting membrane potentials and capacitance of patched neurons in the four groups. (d-e) mEPSC tracings are shown in neurons transfected with NC, Endo2 siRNA, GFP, and Endo2-GFP. (f) Histogram plots of mEPSC frequency and amplitude in neurons transfected with NC and Endo2 siRNA. *n* = 16 cells, 3 cultures from 6 SD rats. (g) Histogram plots of mEPSC frequency and amplitude in neurons transfected with GFP and Endo2-GFP. *n* = 16 cells, 3 cultures from 6 SD rats.

In the resting state, postsynaptic AMPARs rapidly insert into and evacuate from the membrane at almost the same speed, with no presynaptic transmitter being involved. The process of removal of AMPARs from the membrane is called constitutive AMPAR endocytosis. To reveal the effect of endophilin2 on AMPAR endocytosis, the impact on constitutive AMPAR endocytosis should first be determined. In cultured hippocampal neurons, endogenous endophilin2 was knocked down (the efficiency and the specificity have been determined previously [36]) or overexpressed by transfection, and then AMPAR-mediated mEPSCs were recorded. As shown in Figure 1(a), healthy cells with the same membrane capacitance and resting potential (AP) were selected to determine the intrinsic electrophysiological properties of transfected neurons (Figures 1(b) and 1(c)). Compared with the NC group, the genetic silencing of endophilin2 showed no difference on the frequency and amplitude of mEPSCs (Figures 1(d) and 1(f)). Moreover, overexpression of endophilin2 also resulted in no changes to the mEPSCs (Figures 1(e) and 1(g)). These findings suggest that the alteration of endophilin2 expression does not affect AMPAR constitutive endocytosis.

### 3.2. Knockdown but Not Overexpression of Endophilin2 Resists Oligomeric A*β*-Induced AMPAR Dysfunction

**Figure 2:**
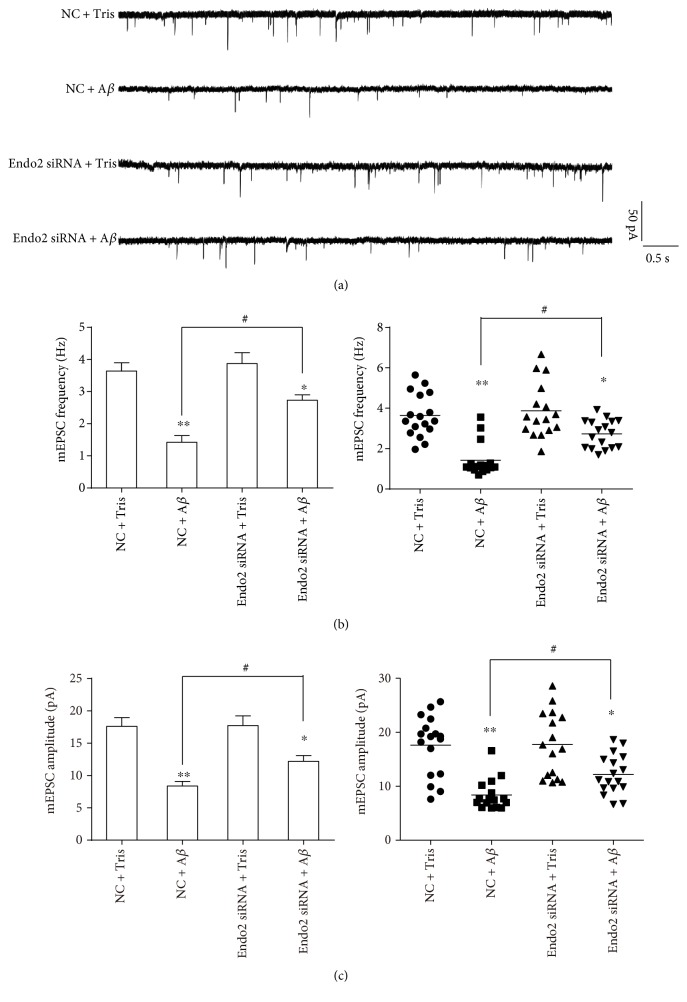
The electrophysiology of endophilin2 knockdown neurons processed by oligomeric A*β*. (a) mEPSC tracings are shown in control and oligomeric A*β*-treated neurons transfected with NC and Endo2 siRNA. (b) Histogram plots and scatterplots of mEPSC frequency in control and oligomeric A*β*-processed neurons transfected with NC and Endo2 siRNA, *n* = 17 cells in NC + Tris and Endo2 SiRNA + A*β* groups, *n* = 16 cells in NC + A*β* and Endo2 SiRNA + Tris groups, 4 cultures from 8 SD rats, ^∗^*P* < 0.05, ^∗∗^*P* < 0.005, and ^#^*P* < 0.005. (c) Histogram plots and scatterplots of mEPSC amplitude in control and in oligomeric A*β*-processed neurons transfected with NC and Endo2 siRNA, *n* = 17 cells in NC + Tris and Endo2 SiRNA + A*β* groups, *n* = 16 cells in NC + A*β* and Endo2 SiRNA + Tris groups, 4 cultures from 8 SD rats, ^∗^*P* < 0.05, ^∗∗^*P* < 0.005, and ^#^*P* < 0.005.

**Figure 3:**
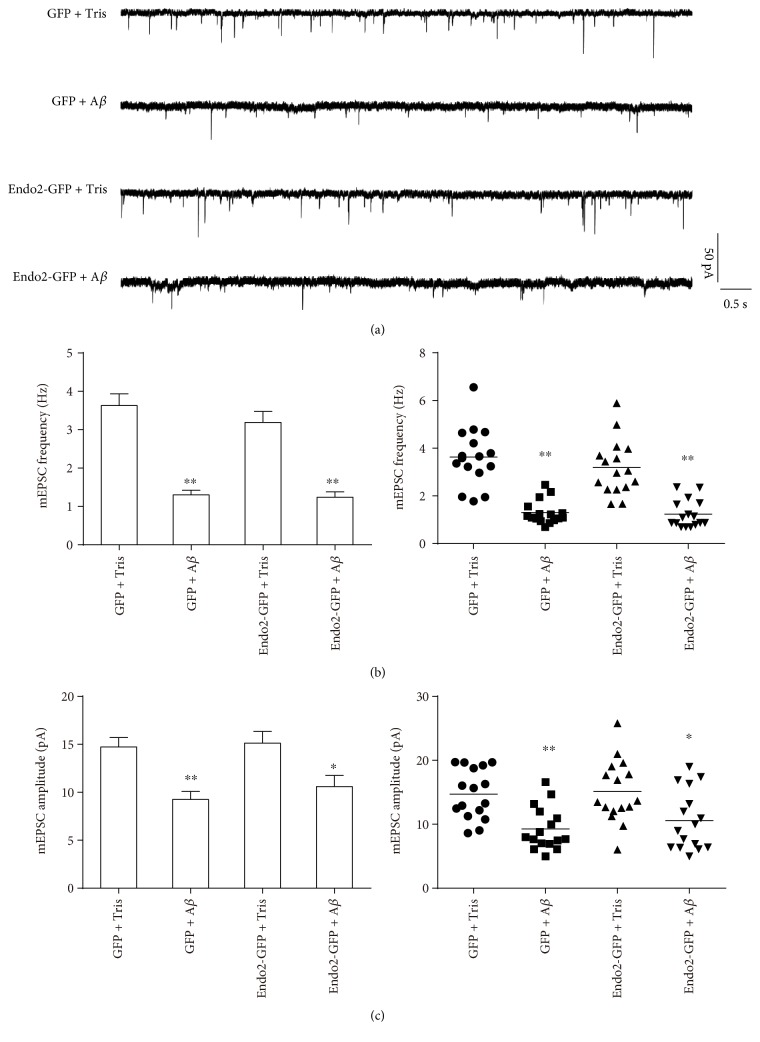
The electrophysiology of endophilin2 overexpression in neurons processed by oligomeric A*β*. (a) mEPSC tracings are shown in control and oligomeric A*β*-processed neurons transfected with GFP and Endo2-GFP. (b) Histogram plots and scatterplots of mEPSC frequency in control and oligomeric A*β*-processed neurons transfected with GFP and Endo2-GFP, *n* = 16 cells, 4 cultures from 8 SD rats, ^∗∗^*P* < 0.005. (c) Histogram plots and scatterplots of mEPSC amplitude in control and oligomeric A*β*-processed neurons transfected with GFP and Endo2-GFP, *n* = 16 cells, 4 cultures from 8 SD rats, ^∗^*P* < 0.05, ^∗∗^*P* < 0.005.

Soluble A*β* oligomers can weaken AMPAR function. When brain slices were incubated with 1 *μ*M A*β*1-42 oligomers, both the frequency and amplitude of AMPAR-induced mEPSCs were reduced significantly [44]. A*β*1-42 oligomers also suppress the AMPAR function in hippocampal slices, inducing synaptic inhibition [18, 19]. To explore the role of endophilin2 in oligomeric A*β*-induced AMPAR dysfunction, cultured DIV8 hippocampal neurons were transfected with NC or endophilin2 siRNA fragments for 48 h and then incubated with 1 *μ*M A*β*1-42 oligomers for another 24 h. As shown in Figure 2, the administration of oligomeric A*β* reduced both the frequency and amplitude of mEPSCs. Moreover, when endophilin2 was silenced, the oligomeric A*β*-reduced mEPSCs recovered (Figure 2), suggesting that the involvement of endophilin2 in the AMPAR dysfunction induced by A*β* oligomers and that the genetic knockdown of endophilin2 can resist oligomeric A*β*-induced AMPAR dysfunction. Conversely, we wondered whether overexpression of endophilin2 would exacerbate oligomeric A*β*-induced AMPAR dysfunction. Neurons were therefore transfected with GFP or GFP-endophilin2- (Endo2-GFP-) encoding plasmids under oligomeric A*β*1-42 treatment, and mEPSCs were then recorded. The addition of A*β* oligomers significantly and consistently decreased the frequency and amplitude of mEPSCs. However, overexpression of endophilin2 showed no changes in mEPSCs in comparison with the GFP group (*P* > 0.05, Figure 3). These results reveal that endophilin2 silencing relieves oligomeric A*β*-induced AMPAR dysfunction but that overexpression of endophilin2 has no effect on it.

### 3.3. Endophilin2 Interacts with GluA1

**Figure 4:**
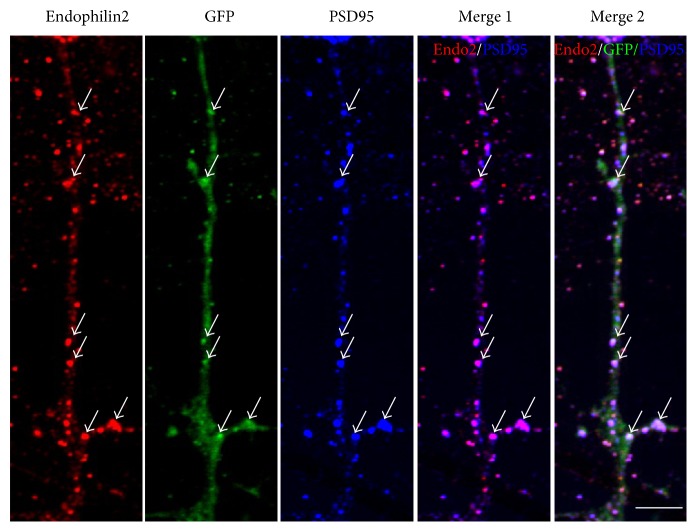
Endophilin2 localizes to postsynaptic sites in mature neurons. DIV14 hippocampal neurons expressing the green fluorescent protein (GFP) as a volume marker were immunostained with antibodies to endophilin2 (red) and PSD95 (blue). Merge 1 shows the endophilin2 colocalization with PSD95 and merge 2 the three proteins. Arrows indicate the colocalization part as indicated. The images shown are representative confocal microscopy images. Scale bar, 5 *μ*m.

To explore the relationship between endophilin2 and AMPARs, we firstly determined the distribution of postsynaptic endophilin2 in cultured hippocampal neurons. GFP-encoding plasmids were transfected to reveal dendrites and spines, and then endogenous endophilin2 and PSD95 proteins were immunostained. As shown in Figure 4, endophilin2 (red) proteins were scattered along dendrite, especially in spine position with stronger red staining; purple signals can be found when merged with PSD95 (blue) as indicated. Furthermore, a part of endophilin2 colocalized with PSD95 proteins revealed by merged white signal from red endophilin2, GFP, and blue PSD95 proteins. The immunostaining results indicated that endophilin2 was abundantly distributed in postsynaptic parts, which is a finding consistent with previous reports [35].

**Figure 5:**
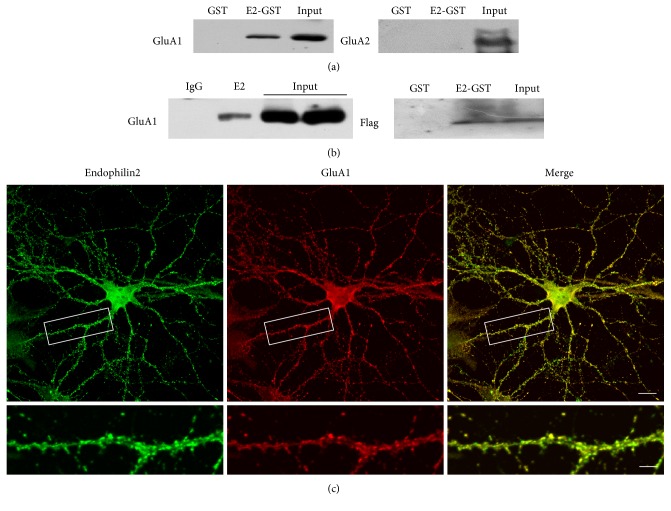
Endophilin2 colocalizes with GluA1. (a) GST-endophilin2 binds to GluA1 not GluA2. GST-tagged endophilin2 fragments were incubated with 1-month-old Sprague Dawley rat brain lysates. Input and bound proteins were analyzed by immunoblotting with antibodies against GluA1 and GluA2. (b) Lysates from 1-month-old Sprague Dawley rat brain were subjected to coimmunoprecipitation with endophilin2 antibody and then subjected to Western blot analysis with GluA1 antibody (left). GST-tagged endophilin2 fragments were incubated with the lysates of HEK293 cells with GluA1-C-flag overexpression (right). (c) Immunostaining of DIV14 hippocampal cultured neurons shows endogenous endophilin2 localized with GluA1. Scale bars are 20 *μ*m and 5 *μ*m in the magnified dendrite. For pulldown assay, we used rat brain lysates, *n* = 4 from 4 SD rats.

The next procedure involved determination of the potential physical interactions between endophilin2 and AMPAR subunits. We constructed GST-endophilin2 plasmids and purified the proteins. Brain lysates of 1-month-old rats were incubated with GST or GST-endophilin2 proteins to perform GST pulldown. The results showed that GST-endophilin2 interacted with GluA1 subunits but not GluA2 (Figure 5(a)). Moreover, a coimmunoprecipitation experiment showed that endophilin2 antibody markedly precipitated endogenous GluA1 proteins in comparison with normal IgG (Figures 5(b), left panel). We next asked whether endophilin2 would interact with the cytoplasmic part of GluA1. By the GST pulldown assay in HEK293 cells, overexpressed Flag-GluA1 C-terminal proteins were detected in the sediments of GST-endophilin2 proteins. Moreover, the immunostainings showed the colocalization of endophilin2 and GluA1 in dendrites, of scattered distribution (Figure 5(c)). These data indicates that endophilin2 protein interacts with one subunit of AMPAR: GluA1.

### 3.4. Endophilin2 Interacts with GluA1 to Regulate Oligomeric A*β*-Induced AMPAR Dysfunction

**Figure 6:**
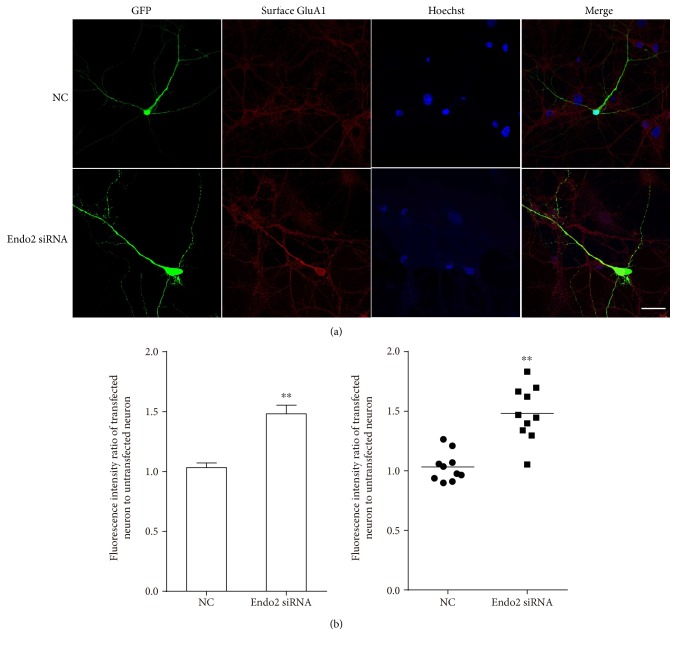
The expression levels of surface GluA1 in endophilin2 knockdown neurons treated by oligomeric A*β*. (a) Hippocampal cultured neurons transfected with NC and Endo2 siRNA immunostained with surface GluA1. Scale bar is 20 *μ*m. (b) The fluorescence intensity of surface GluA1 in cytoplasm transfected with Endo2 siRNA or NC was normalized to neighboring untransfected neurons. *n* = 10 cells, 3 cultures from 6 SD rats, ^∗∗^*P* < 0.005.

After determining the interaction between endophilin2 and GluA1, we considered whether this interaction would mediate the endocytosis of GluA1. Neurons transfected with NC or endophilin2 siRNA fragments were treated with 1 *μ*M A*β*1-42 oligomers for 24 h to stimulate the endocytosis of AMPAR, and after fixation with 4% paraformaldehyde, the surface GluA1 proteins were stained. The results showed that after the oligomeric A*β* treatment, the signals from surface GluA1 proteins in the control group were weak, whereas in the endophilin2 knockdown group, the signals from the surface GluA1 proteins were strong (Figure 6(a)). The statistical data are shown in Figure 6(b). These results suggest that genetic knockdown of endophilin2 may weaken the interaction with GluA1, which then inhibits the endocytosis of GluA1 and relieves oligomeric A*β*-induced AMPAR endocytosis.

## 4. Discussion

In this study, we demonstrate that endophilin2 does not contribute to constitutive AMPAR endocytosis. Oligomeric A*β* induces AMPAR dysfunction; endophilin2 knockdown can resist the process, while endophilin2 overexpression cannot. Moreover, endophilin2 interacts with GluA1, a subunit of AMPAR, to regulate oligomeric A*β*-induced AMPAR endocytosis. Increasing evidence has shown that oligomeric A*β* plays a critical role in the cognitive impairment of AD patients. During the early processes of AD, A*β* oligomers can induce the loss of postsynaptic AMPARs, leading to LTD induction and LTP inhibition [25, 45]. Therefore, the prevention of synaptic damage could be an effective strategy to avoid AD-related cognitive disorders and improve learning and memory ability.

A*β* is an enzymatic product and exists in two main forms, A*β*1-42 and A*β*1-40, with the A*β*1-42 oligomers believed to have greater neuronal toxicity [20, 21, 28]. Our results on hippocampal neurons cultured with 1 *μ*M A*β*1-42 oligomers showed that the administration of oligomeric A*β* significantly decreased the frequency and amplitude of mEPSCs (Figure 2), indicating a marked dysfunction of AMPARs. Furthermore, the knockdown of endophilin2 significantly relieved the decreased mEPSCs (Figure 2), indicating that the rescued oligomeric A*β* induced AMPAR dysfunction. Overendocytosis of subunits of AMPAR from the postsynaptic membranes [27, 28], or failure of insertion of them into postsynaptic membranes [46, 47], is the main mechanism for oligomeric A*β*-induced AMPAR dysfunction. Promoting the insertion of AMPARs or suppressing endocytosis could therefore be important ways to improve AMPAR function. Recently, applications of neurogranin and leptin have shown significant improvement to the inhibition of oligomeric A*β*. Neurogranin can activate CaMKII to promote the insertion of GluA1 into postsynaptic membranes, leading to an increased AMPAR current and restoration of A*β*-induced LTP deficit [48]. Leptin, via the inhibition of GluA1 endocytosis, prevents hippocampal synaptic disruption and inhibits LTD facilitation [39]. Our results showed that genetic knockdown of endophilin2 suppressed GluA1 endocytosis (Figure 6), with the amount of surface GluA1 in the knockdown group being 1.5-fold that of the control group, thus indicating the important role of endophilin2 in AMPAR endocytosis. Fluorescence staining showed that endophilin2 siRNA significantly suppressed the surface GluA1 endocytosis, while the changes in mEPSCs may seem modest. This may due to that A*β* oligomers reduce the surface of GluA1 and GluA2 [27], while endophilin2 only shows impact on GluA1.

When endophilin2 is overexpressed, no promoting effect of AMPAR endocytosis is observed. Additionally, neither overexpression nor silencing of endophilin2 showed any impact on the constitutive endocytosis of AMPARs (Figure 1). During the process of oligomeric A*β*-mediated AMPAR endocytosis, decreasing the expression of endophilin2 affected the endocytosis but increasing it did not, thereby indicating that the physiological concentration of endophilin2 can satisfy AMPAR endocytosis. In this study, we demonstrate the impact of endophilin2 knockdown on the AMPAR current in cultured neurons; whether or not the inhibition of endophilin2 can improve LTP and suppress the facilitation of LTD in AD requires further exploration.

Endophilins are a cytosolic protein distributed within the soma, dendrites, and spines. Previous studies and our former research have mainly focused on the presynaptic endophilin proteins during synaptic vesicle endocytosis. In the current study, our immunostaining clearly shows the distribution of endophilin2 on dendrites, especially on spines, where it is colocalized with PSD95 proteins, suggesting an abundant expression in postsynaptic parts. This is consistent with the results from Chowdhury and colleagues [35]. The immunostaining showed that GluA1 colocalized with endogenous endophilin2 (Figure 5), and, moreover, the pulldown and immunoprecipitation results showed that endophilin2 interacted with GluA1 but not GluA2 (Figure 5). Endophilins are involved in AMPAR endocytosis, as was first reported by Chowdhury and colleagues [35]. In their study, endophilins and dynamins, both as assistant proteins, formed complexes with the immediate early gene Arc/Arg3.1 to mediate AMPAR endocytosis. However, none of these proteins showed a direct interaction with AMPARs. Here, we have provided evidence of endophilin2 interacting with AMPARs. Endophilin2 has been confirmed as a calcium-binding protein, acting as a calcium sensor in presynaptic vesicle endocytosis [37]. Thus, endophilin2 may act either as a receptor-binding protein to mediate endocytosis or as a sensor to initiate endocytosis. Slow calcium influx is the trigger for AMPAR endocytosis; however, how calcium triggers such AMPAR endocytosis is an interesting question, which will be a subject of investigation in our future studies.

In summary, we show for the first time that endophilin2 interacts with AMPARs to regulate oligomeric A*β*-mediated AMPAR endocytosis in primary cultured hippocampal neurons. Genetic silencing of endophilin2 inhibits GluA1 endocytosis to alleviate oligomeric A*β*-mediated AMPAR dysfunction. This work indicates possible directions for drug development for AD therapy to prevent or even reverse the loss of AMPAR, which could help to improve learning and memory in AD patients.

## 5. Conclusion

Oligomeric A*β* plays an important role in mediating the cognitive deficits in AD, as A*β* oligomers can induce the loss of postsynaptic AMPARs. Interference with endophilin2 can alleviate the decreased AMPAR function, whereas overexpression of endophilin2 has no effect on the decreased AMPAR function induced by A*β* oligomers. Endophilin2 interacts with GluA1, a subunit of AMPAR, to regulate oligomeric A*β*-induced AMPAR endocytosis.
